# Testing the contribution of dispersal to microbial succession following a wildfire

**DOI:** 10.1128/msystems.00579-23

**Published:** 2023-09-25

**Authors:** Kristin M. Barbour, Claudia Weihe, Kendra E. Walters, Jennifer B. H. Martiny

**Affiliations:** 1 Department of Ecology and Evolutionary Biology, University of California-Irvine, Irvine, California, USA; 2 Biology Department, Reed College, Portland, Oregon, USA; Ashley Shade, CNRS Delegation Alpes, Lyon, Rhône-Alpes, France

**Keywords:** microbial dispersal, wildfire, succession

## Abstract

Given increased wildfire activity, there is growing interest in understanding the drivers of microbial succession after fire. Dispersal may be especially important to post-fire succession as biotic communities can be more susceptible to invasion following a disturbance. Here, we experimentally manipulated dispersal into disturbed leaf litter communities collected following a wildfire and tracked bacterial and fungal dispersal assemblages over time. We show that the identity and source of microbes immigrating into the soil surface post-fire change across time with seasonal shifts and the reemergence of aboveground vegetation. Further, dispersal significantly contributed to the reassembly of leaf litter microbial communities after the fire, producing an increasingly distinct assembly trajectory. The effect of dispersal on α-diversity and β-diversity was ecosystem dependent but, unexpectedly, influenced bacterial and fungal communities in a similar manner within ecosystems. Collectively, these results demonstrate that dispersal explicitly alters the course of microbial community succession following a wildfire and may impact bacteria and fungi in parallel ways, despite differing in traits expected to alter dispersal patterns.

**IMPORTANCE:**

Identifying the mechanisms underlying microbial community succession is necessary for predicting how microbial communities, and their functioning, will respond to future environmental change. Dispersal is one mechanism expected to affect microbial succession, yet the difficult nature of manipulating microorganisms in the environment has limited our understanding of its contribution. Using a dispersal exclusion experiment, this study isolates the specific effect of environmental dispersal on bacterial and fungal community assembly over time following a wildfire. The work demonstrates the potential to quantify dispersal impacts on soil microbial communities over time and test how dispersal might further interact with other assembly processes in response to environmental change.

## INTRODUCTION

Dispersal, or the movement of organisms across space, has been recognized as a fundamental mechanism influencing microbial community assembly (1, 2). Like other biological processes (e.g., selection, speciation, and drift), the contribution of dispersal to community assembly can vary based on contemporary and historical conditions, such as after a disturbance (3, 4). Wildfire is one disturbance that has rapidly increased in frequency and intensity over the last few decades, particularly in drought-prone regions such as the Southwestern United States (5, 6). Given that fire activity is predicted to continue increasing (7, 8), there is considerable interest in understanding how ecological communities respond to and recover from fire, especially in grasslands were a majority of annual global fires occur (9). Historically, researchers have focused on the secondary succession of plant communities, but there are growing efforts to understand the assembly of soil microbial communities (10) due to their role in post-fire nutrient cycling (11, 12) and plant restoration (13).

Wildfires can alter surface soil microbial communities, including those in leaf litter, directly through heating and indirectly by altering the physical and chemical properties of bulk soil and leaf litter, such as through the incomplete combustion of organic matter (14, 15). The specific effect of wildfire on microbial communities is highly variable, with some studies reporting no change in α-diversity (16 – 18) and others reporting effects on composition, abundance, and diversity that last years (19) to decades (20). This variability is likely due to differences in fire severity, soil type, pre-fire microbial community, and sampling methods (i.e., soil depth and timing post-fire) between studies. Despite these inconsistencies, some general patterns do emerge. For instance, wildfires typically reduce overall microbial abundance and richness in the surface soil (21). Additionally, fungi are generally more sensitive to fire than bacterial communities, perhaps due to a lower heat tolerance or the death of plants associated with mycorrhizal fungi (21, 22). Post-fire surveys have also shown that fire can select for pyrophilous or “fire-loving” microbes such as fungi in the genera *Pyronema* and bacteria in the spore-forming phylum *Firmicutes*, or in the genus *Massilia* (phylum *Proteobacteria*) (23 – 26).

Given that wildfire can dramatically lower microbial abundance and diversity, dispersal may be especially important to post-fire succession, defined here as the sequential manner by which communities change over time following a disturbance (27). Dispersal can influence community reassembly in numerous ways. For instance, dispersal can reintroduce (or rescue from lowered abundances) taxa more abundant in the pre-fire community (28, 29). Dispersal can also facilitate the arrival of novel taxa that are better suited for the post-fire conditions and, thus, outcompete resident taxa (30, 31). Alternatively, high dispersal rates can introduce mal-adapted individuals, potentially impeding community resilience (32 – 34). Finally, dispersal can alter overall β-diversity, or the variance in composition between local communities, depending on how variable the assemblage of dispersing microbes is across a landscape (4, 35). In sum, there are a variety of ways that microbial dispersal is expected to influence post-fire succession; however, the specific effects of dispersal have not been assessed by experimentally manipulating dispersal in an environmental community.

**FIG 1:**
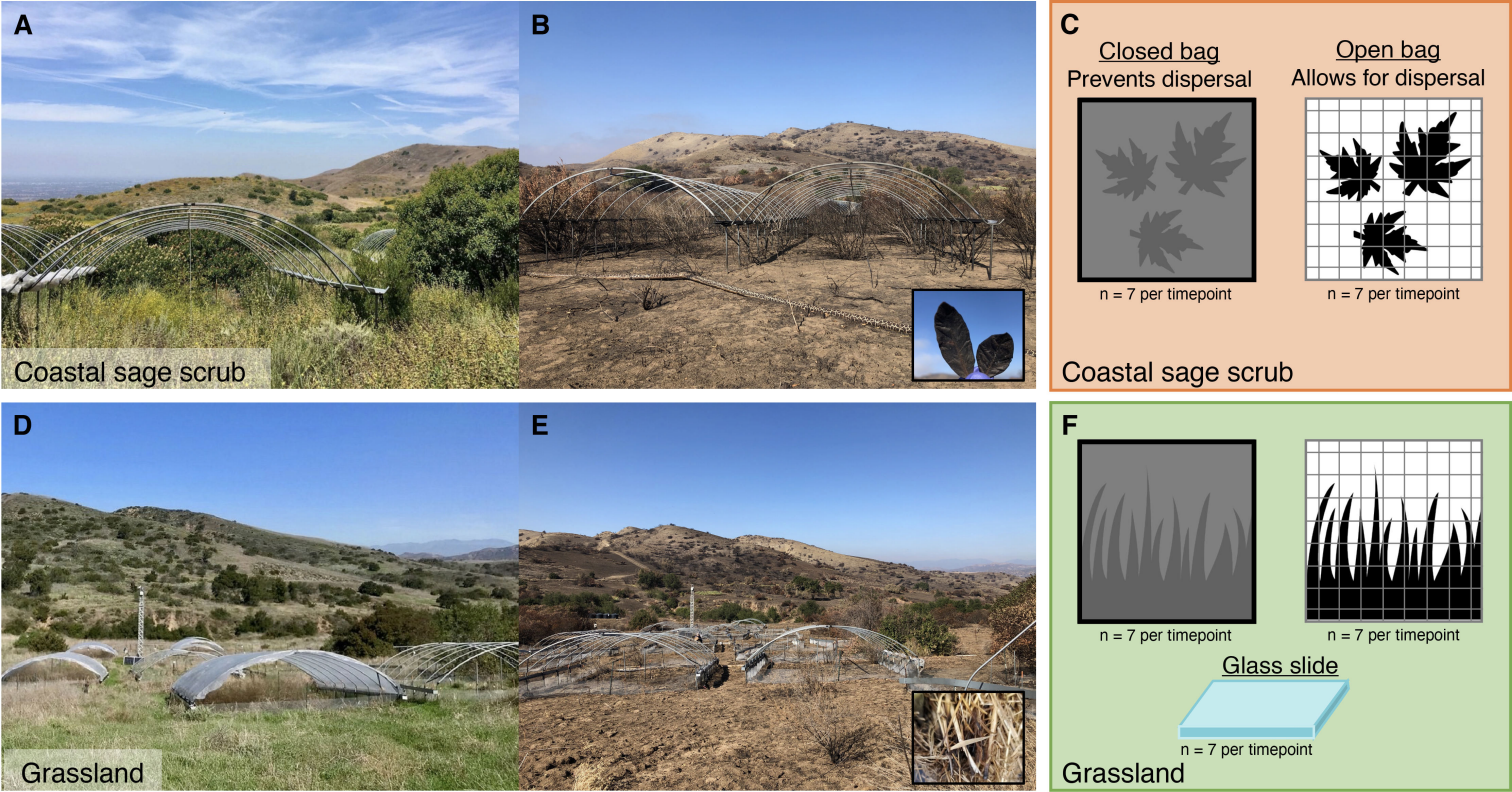
Loma Ridge coastal sage scrub (CSS) (**A**) before and (**B**) after the Silverado Fire in 2020. (B, inset) Burned CSS leaf litter collected after the wildfire. (**C**) Leaf litter dispersal treatment bags deployed into the CSS in November 2020. Closed dispersal bags are made of 0.22-µm nylon mesh, preventing microbial cells from moving in or out of the bag. Open dispersal bags are made of 2-mm window screen, allowing cells to disperse into the bag. Loma Ridge grassland (**D**) before and (**E**) after the Silverado Fire. (E, inset) Burned grassland leaf litter collected after the wildfire. (**F**) Burned leaf litter dispersal treatment bags and glass slides deployed into the grassland in November 2020. Glass slides were not deployed in the CSS due to resource constraints.

Here, we investigated the impacts of dispersal on microbial communities in leaf litter, the topmost layer of soil, following a vegetation fire in two adjacent ecosystems in Southern California, a semi-arid grassland and coastal sage scrub (CSS). Fueled by hot, dry summers and strong Santa Ana winds, wildfires are common in these Mediterranean-type ecosystems with six fires recorded at our experimental field site since the beginning of the 20th century (1914, 1948, 1967, 1998, 2007, and 2020) (36). To test the influence of dispersal on the post-fire succession of leaf litter microbial communities, we constructed bags which either permitted (“open” bags, 2-mm window screen) or prevented (“closed” bags, 0.22-µm nylon) microbial cells from immigrating in or out. The bags were filled with either sterile glass microscope slides (grassland only) or charred leaf litter collected after the wildfire in 2020 (Fig. 1; Fig. S1). Glass microscope slides capture microbial cells immigrating into the surface soil while restricting cell growth by not providing an energy source (37). In comparison, the charred leaf litter allowed us to assess the role of post-fire dispersal on fungal and bacterial community succession in the field.

We hypothesized that dispersal impacts the succession of post-fire leaf litter communities. To address this hypothesis, we asked two questions:

1. What is the identity and source of microbes (both bacteria and fungi) dispersing into the soil surface following a fire? We expected that dispersal from air and exposed bulk soil (via wind and rain) would be the dominant dispersal source into the leaf litter layer post-fire. However, we also anticipated that the composition of dispersing propagules would change over time as vegetation, a key source of dispersal into the soil surface at this site pre-fire (37), recovered.
2. How does dispersal influence (i) composition of bacterial and fungal communities and (ii) specifically, their abundance and α-diversity and β-diversity during post-fire succession? We predicted that dispersal would quickly alter community composition post-fire, resulting in an alternative assembly trajectory whereby over time, communities will become increasingly dissimilar to communities closed to dispersal. We also predicted that exposure to dispersal would increase α-diversity after the fire, but especially for fungi because of their greater sensitivity to wildfire than bacteria. Similarly, we expected that dispersal would increase bacterial and fungal β-diversity during post-fire succession as the vegetation recovered in patches.

## RESULTS

### The microbial dispersal assemblage changes over time

**FIG 2:**
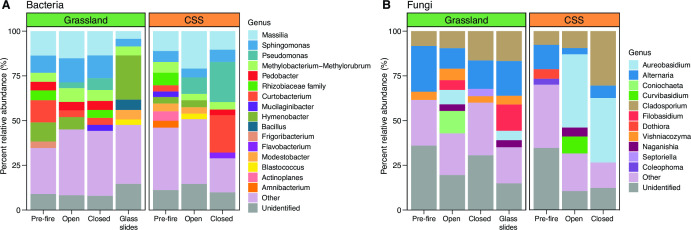
Composition of (**A**) bacterial and (**B**) fungal genera on pre-fire and burned (open and closed) leaf litter from the grassland and CSS and glass slides (grassland only) for all timepoints. Pre-fire composition is represented by the average community composition on leaf litter samples collected between 2016 and 2018 from this field site (38). Open and closed composition is represented by the average community composition of all burned leaf litter samples open and closed to dispersal, respectively, collected for this experiment. Glass slide composition is represented by the average community composition on all glass slides open to dispersal. “Other” genera represent all classified genera below 3% relative abundance.

To characterize the microbial propagules dispersing into the soil surface (hereafter, the “dispersal assemblage”), we assessed the taxonomic composition (bacteria and fungi) and the abundance (bacteria only) of the cells captured on the glass slides in the grassland only. The dispersal assemblage was differentiated from burned leaf litter communities by a higher relative abundance of the bacterial genus *Hymenobacter* and fungal genus *Filobasidium* (Fig. 2; Table S1; similarity percentages [SIMPER] analysis).

**FIG 3:**
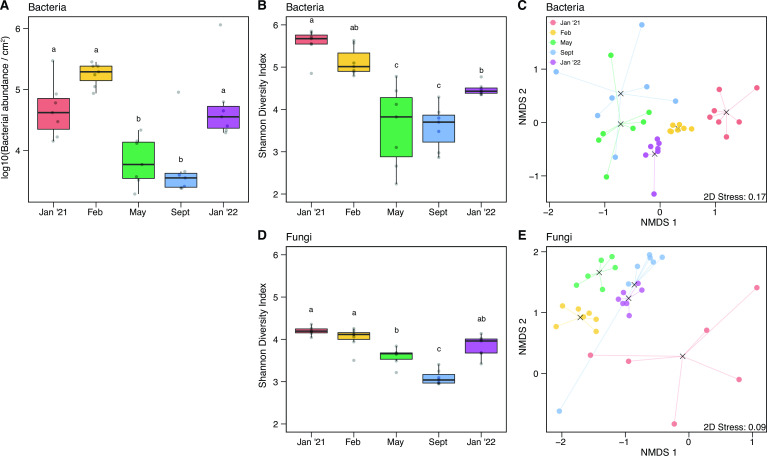
(**A**) Abundance, (**B**) diversity, and (**C**) non-metric multidimensional scaling (NMDS) ordination of bacteria dispersing onto the glass slides over time. (**D**) Diversity and (**E**) NMDS ordination of fungi dispersing onto the glass slides over time. All glass slide samples were collected from the grassland only.

Since abiotic and biotic properties of the landscape changed throughout the duration of the experiment as seasons shifted and aboveground vegetation recovered, we specifically tested if the dispersal assemblage changed over time. The abundance of immigrating bacterial cells varied over time (Fig. 3A; Table S2; analysis of variance [ANOVA]: *P* < 0.001) and was higher during the wet season in January and February (post hoc comparison: *P* < 0.05). Bacterial diversity (Shannon diversity index) also changed across time (ANOVA: *P <* 0.001) and showed a similar pattern as abundance, peaking during the wet months (Fig. 3B; Table S3; *P* < 0.001). Further, the composition of the bacteria dispersing into the soil surface also changed (Fig. 3C; Table S4; permutational multivariate analysis of variance [PERMANOVA]: *P* ≤ 0.001). Initially, *Actinobacteria* dominated the dispersal assemblage (January 2021 abundance: 50.6%). However, by the end of the experiment, the majority of dispersing bacteria were from the phylum *Bacteroidetes* (January 2022 abundance: 54.0%). This broad shift in composition was driven by a 134-fold increase in the relative abundance of the genus *Hymenobacter* (phylum *Bacteroidetes*) from 0.29% at the first timepoint to 39% at the final timepoint (Fig. S2A). Along with taxonomic changes, β-diversity, or the compositional variability of immigrating bacteria among sampling locations, also changed across time. Specifically, the composition of bacteria dispersing into the soil surface was most variable across the landscape during the dry season (May and September) (Fig. 3C; PERMDISP post hoc pairwise comparisons: *P* < 0.05).

Following the same pattern as bacterial diversity, the diversity of fungi dispersing into the soil surface varied over time (Fig. 3D; Table S5; ANOVA: *P* < 0.001) and was generally higher during the wet season. (We did not assess fungal abundance on the glass slides, so we cannot compare to bacterial abundance.) The composition of fungi immigrating onto the glass slides also varied temporally (Fig. 3E; Table S6; PERMANOVA: *P* < 0.001), with the first post-fire samples (January 2021) being quite distinct and more variable in composition compared to later timepoints (PERMDISP post hoc pairwise-comparisons: *P* < 0.05). Throughout the course of the experiment, the fungal dispersal assemblage was dominated by the phyla *Ascomycota* and *Basidiomycota*, but notably, there was a threefold increase in the relative abundance of the genus *Alternaria* (phylum *Ascomycota*) from 9.5% to 28% from the first to the final timepoint (Fig. S2B). *Alternaria* also dominates the unburned leaf litter fungal community at this field site (38).

### Sources of dispersing microbes

**FIG 4:**
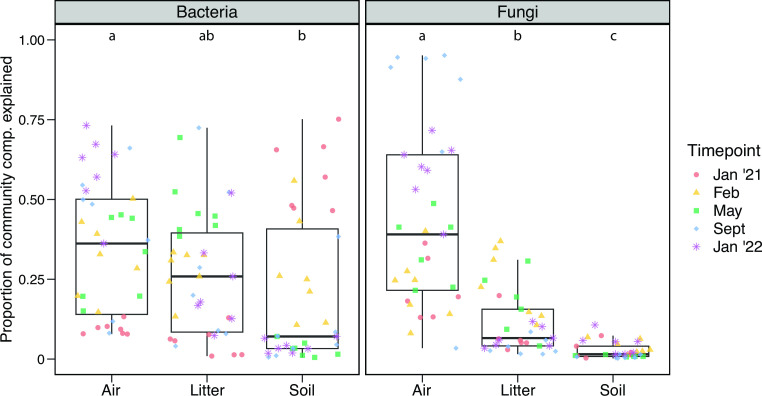
Proportion of bacterial and fungal community composition on the glass slides attributable to different dispersal source communities (air samples, environmental leaf litter, and surface bulk soil). Color and shape indicate the timepoint from which the sample was collected from the grassland. Letters indicate significant pairwise differences between dispersal sources (Dunn’s multiple comparison post hoc test using Bonferroni correction).

To investigate where bacteria and fungi on the glass slides were immigrating from, we sampled microbial communities from three potential dispersal sources (air, surrounding leaf litter, and soil) collected at each timepoint. Bacterial and fungal composition were significantly different between all the three dispersal sources (Fig. S3; PERMANOVA post hoc pairwise comparisons: *P* < 0.001). A SourceTracker analysis found that these sources varied in their contribution to the bacterial and fungal dispersal assemblages found on the glass slides (Fig. 4; Kruskal-Wallis: *P* < 0.01 in both cases). The largest proportion of the bacterial dispersal assemblage could be traced back to air and environmental leaf litter (34% and 26%, respectively), while dispersal from air alone explained the greatest proportion of the fungal community on the glass slides (42%). Against our expectations, dispersal from bulk soil contributed a smaller amount to the overall bacterial and fungal dispersal assemblages (20% and 3%, respectively); however, it explained the largest proportion of the bacterial community on the glass slides in January 2021, prior to the reemergence of vegetation (Fig. 4, red points).

### Wildfire effects on the microbial leaf litter community

In addition to characterizing the microbes dispersing onto the soil surface, we also assessed how dispersal influenced the succession of microbial communities on burned leaf litter. To validate that the leaf litter communities were disturbed by the fire, we compared microbial composition on the burned litter collected after the Silverado Fire in 2020 with pre-fire litter collected at the same field site between 2016 and 2018 (38). Post-fire bacterial and fungal compositions were significantly different from pre-fire communities in both ecosystems, regardless of the dispersal treatment (Fig. 2; PERMANOVA post hoc pairwise comparisons: *P* ≤ 0.001 all cases). This result supports previous findings showing these leaf litter communities were altered by the wildfire (39). Overall, burned leaf litter was characterized by a higher relative abundance of the bacterial genus *Pseudomonas* and lower relative abundance of the fungal genus *Alternaria* (Fig. 2; Table S7; SIMPER analysis). In particular, the burned CSS leaf litter was dominated by the fungal genus *Aureobasidium*, which showed 18-fold and 16-fold increases in relative abundance in the open and closed bags, respectively, compared to the unburned community (Fig. 2B).

### Dispersal affects microbial succession post-fire

**Fig 5:**
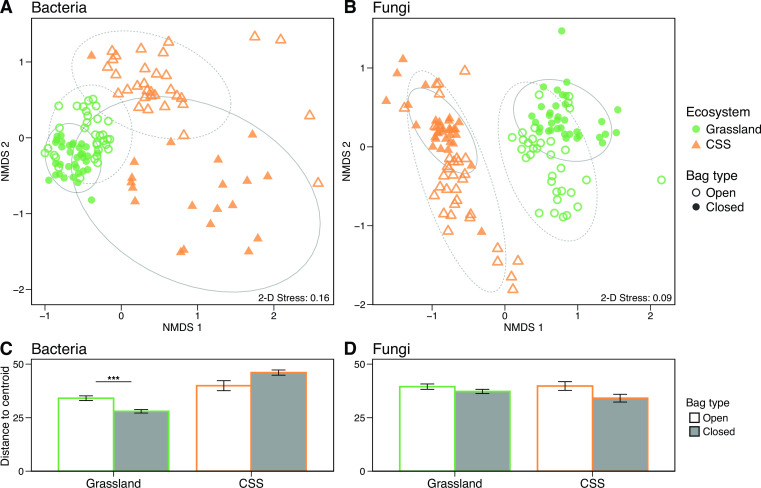
Non-metric multidimensional scaling (NMDS) ordination of leaf litter (**A**) bacterial and (**B**) fungal community composition performed on Bray-Curtis dissimilarities of rarefied and square root transformed operational taxonomic unit tables. Symbol color represents ecosystem type (green, grassland; orange, CSS), and symbol shape represents dispersal bag type (filled, closed; outline, open). Ninety-five percent confidence intervals are shown around each ecosystem by dispersal bag treatment combination as a whole. Solid and dashed lines represent the confidence intervals for the closed bags and open bags, respectively. See Fig. S4 for the same figure colored by timepoint. Average within-group distances and standard errors for (**C**) bacterial and (**D**) fungal community composition (Bray-Curtis dissimilarity). Asterisks denote significant differences in dispersion between open and closed bags within a single ecosystem (***P ≤ 0.001).

To isolate the effect of dispersal on microbial succession after the wildfire, we compared community assembly on burned leaf litter in open and closed litterbags. As we expected, dispersal significantly contributed to the post-fire succession of microbial communities. In both ecosystems, bacterial and fungal compositions were affected by the dispersal treatment (Fig. 5; Tables S4 and S6; PERMANOVA: *P* ≤ 0.001 in all cases). Overall, dispersal had a greater impact on the post-fire assembly of the bacterial community, explaining a larger proportion of compositional variation (18% and 34% in the grassland and the CSS, respectively) compared to the fungal communities (15% and 21%) (Fig. S4; Tables S4 and S6). In the grassland, leaf litter communities exposed to dispersal were represented by a higher relative abundance of the bacterial genera *Massilia* and *Hymenobacter* as well as the fungal genus *Coniochaeta* (Fig. 2; Table S8; SIMPER analysis). CSS leaf litter communities in the absence of dispersal were characterized by a greater relative abundance of the bacterial genus *Curtobacterium* and fungal genus *Cladosporium* compared to the open bags (Fig. 2; Table S8; SIMPER analysis).

Dispersal also affected how bacterial communities assembled over time (Fig. S5A; Table S4; PERMANOVA: bag type by timepoint interaction, *P* ≤ 0.001 in both ecosystems). Bacterial composition did not initially differ between the dispersal treatments in January 2021, 3 months after the fire (post hoc pairwise comparison: *P* > 0.05). However, as we expected, the effect of dispersal on bacterial assembly increased with time in both ecosystems, such that community composition was most dissimilar between the open and closed bags at the final collection in January 2022, 14 months after the fire.

Exposure to dispersal altered fungal community succession over time in a similar manner (Fig. S5B; Table S6; PERMANOVA: bag type by timepoint interaction, *P* ≤ 0.001 in both ecosystems). Like the bacterial communities, the effect of dispersal increased with time. Fungal composition did not differ between the open and closed bags in either ecosystem until the second collection in February 2021 (post hoc pairwise comparison: *P* < 0.05) and composition were most dissimilar between dispersal treatments toward the end of the experiment (January 2022 in the grassland and September 2021 in the CSS).

### Dispersal differentially affects microbial abundance and α-diversity and β-diversity on leaf litter

**Fig 6:**
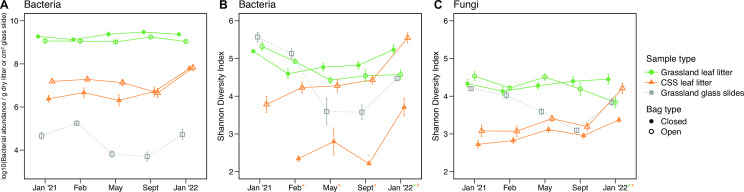
Average leaf litter (**A**) bacterial abundance, (**B**) bacterial diversity, and (**C**) fungal diversity in the open and closed bags from both the grassland and CSS. Sample type is represented by symbol color. Symbol shape indicates bag type. Asterisks on the *x*-axis indicate pairwise significance (*P* < 0.05) between open and closed leaf litter bags by month (Tukey’s honestly significant difference). Asterisk color represents the ecosystem(s) where significance occurred (green, grassland; orange, CSS). Bacterial abundance calculated as per square centimeter of the glass slide and per gram of dry litter for the glass slide and leaf litter samples, respectively.

Exposure to dispersal altered bacterial abundance on the leaf litter but did so in an ecosystem-dependent manner (Fig. 6A; Table S2; ANOVA: ecosystem by bag type interaction, *P* < 0.001). In the grassland, bacterial abundance in the open litter bags was 46% lower than that in the closed bags (open = 1.3 × 10^9^ cells/g dry litter; closed = 2.4 × 10^9^ cells/g dry litter; ANOVA: bag type, *P* < 0.001). In contrast, exposure to dispersal increased bacterial abundance in the CSS leaf litter by 47% (open = 3.1 × 10^7^ cells/g dry litter; closed = 2.1 × 10^7^ cells/g dry litter; *P* < 0.001). Moreover, the effect of dispersal on bacterial abundance in the CSS litter changed over time (bag type by timepoint interaction: *P* < 0.05), whereby the difference in average abundance between the open and closed bags seen during the first 6 months of the experiment was not detectable by September 2021, 11 months after the wildfire.

Dispersal also affected microbial diversity, influencing bacterial and fungal diversity in a similar manner (Fig. 6B and C). Exposure to dispersal decreased bacterial diversity by an average of 3% in the grassland while increasing diversity by 58% on average in the CSS. However, the effect of dispersal on bacterial diversity changed over time in the grassland (Table S3; bag type by timepoint interaction: *P* < 0.001). Specifically, bacterial diversity in the grassland did not differ between the dispersal treatments until the final timepoint when exposure to dispersal decreased bacterial diversity by 12% on average (Fig. 6B). In contrast, bacterial diversity in the CSS was higher in the open bags for the duration of the experiment.

For the fungal communities, exposure to dispersal did not significantly impact overall diversity in the grassland (Table S5; *P* > 0.05), but increased diversity in the CSS by 13% on average (*P* < 0.001). Like the bacterial communities, fungal diversity was only significantly different between the open and closed bags at the final timepoint in both ecosystems (Fig. 6C).

In addition to affecting α-diversity, exposure to dispersal also changed the β-diversity of the leaf litter communities. However, the effect of dispersal on β-diversity varied between ecosystems. Exposure to dispersal increased overall variability in bacterial composition in the grassland (Fig. 5C; PERMDISP: *P* ≤ 0.001) but marginally decreased β-diversity in the CSS (*P* = 0.096) against our expectations.

In contrast, dispersal did not affect variability in fungal composition in the grassland (Fig. 5D; *P* = 0.20) but marginally increased compositional heterogeneity in the CSS (*P* = 0.065). We note that this variation in dispersion may also contribute to the significant compositional differences found between dispersal treatments in both ecosystems (40).

## DISCUSSION

By manipulating dispersal directly (excluding it completely), this study demonstrates that microbial dispersal influences microbial succession in surface soil. Dispersal is important for the succession of bacteria and fungi on leaf litter following wildfire, a disturbance that alters both the soil microbial community and the assemblage of microbes dispersing into the soil surface.

Given that the wildfire removed much of the vegetation and standing leaf litter, we expected that the air and bulk soil would be key sources of microbial dispersal into the leaf litter layer (Q1). This prediction was only partially supported, as air was a key source of immigrating microbes (bacteria and fungi) post-fire, while the bulk soil was less important [although we note that the air community itself likely includes bacteria and fungi previously liberated from other sources such as bulk soil and the phyllosphere (41, 42)]. Further, a previous study conducted at this field site before the fire found that only 4% of the bacteria immigrating into the surface soil were traced back to the bulk soil, compared to 20% here (a fivefold increase post-fire) (37). Increased importance of these dispersal sources post-fire may be due to the fire removing much of the standing vegetation and persistent leaf litter layer and, thus, reducing physical barriers between the air and soil surface. One caveat of this result is that the glass slides may be less likely to capture taxa that disperse by active dispersal mechanisms, such as fungi that move by hyphal growth (37). Additionally, the glass slides may have selected for taxa with greater resistance to degradation from UV radiation, moisture stress, and nutrient-poor conditions that more closely mirror conditions previously experienced by aerial dispersers. For instance, *Hymenobacter*, the most abundant bacterial genera captured on the glass slides at later timepoints, is a common atmospheric bacterium that displays resistance to radiation (43, 44).

In addition to identifying the key sources of dispersal onto the soil surface, we also characterized the identity of the dispersing propagules (Q1). As we expected, the composition of fungi and bacteria dispersing into the grassland leaf litter layer shifted over time. These temporal changes in the dispersal assemblage may be due to the post-fire plant succession. However, we cannot entirely disentangle how much of this temporal variation was due to the wildfire effects on the landscape versus seasonal shifts in precipitation, wind, and other meteorological factors that alter dispersal patterns across the landscape (45, 46).

Although we cannot trace specific taxa from the dispersal assemblage to the leaf litter, our experiment demonstrates that dispersal alters the successional trajectory of leaf litter microbial communities by impacting composition, abundance, and α-diversity and β-diversity (Q2). The effect of dispersal on specific taxa on leaf litter was highly variable. For instance, exposure to dispersal negatively impacted some taxa, such as the bacterial genus *Curtobacterium* and the fungal genus *Cladosporium.* Both taxa displayed relatively higher abundance in the closed CSS leaf litter communities, indicating that dispersing taxa compete with these taxa. In contrast, the bacterial genus *Massilia* increased in relative abundance in the leaf litter communities exposed to dispersal in both ecosystems. Other taxa showed a minimal response to the dispersal treatment but were greatly impacted by the wildfire. In particular, the fungal genus *Aureobasidium* made up over 36% of the post-fire CSS leaf litter community in both the open and closed bags compared to 2% of the pre-fire community. *Aureobasidium* is not commonly recognized as a pyrophilous fungus; however, the genus was found to be enriched in burned bulk surface soil from a recently burned pine forest (47), suggesting it may have a competitive advantage in post-fire or post-disturbance environments.

Dispersal also impacted leaf litter bacterial and fungal communities in an ecosystem-dependent manner. Previous studies report contrasting effects of dispersal on microbial assembly, but the factors responsible for these differences remain unclear. For instance, exposure to dispersal increased compositional variation (β-diversity) of nectar-inhabiting microbes on flowers (48), while it homogenized bacterial composition on pre-fire leaf litter in this grassland system (49). Still, it is somewhat unexpected to observe differential impacts of dispersal in adjacent vegetation communities. We can think of at least three reasons for this ecosystem dependence in our system. First, the severity of the disturbance may have varied between ecosystems. Specifically, a thinner and more uniform char layer in the grassland suggests the wildfire burned more severely and with greater variability in the CSS than in the grassland at this field site. The effect of wildfire on microbial communities is proportional to the fire severity (24, 50). Thus, leaf litter communities in the CSS may have been differentially susceptible to dispersing microbes compared to those in the grassland. Second, the effect of dispersal may depend on substrate quality, which also differs between ecosystems (51). Given that resource availability can alter invasion success (52, 53), chemical differences between the grass and shrub leaf litter may alter community response to dispersal. Third, the leaf litter may have been exposed to unique dispersal assemblages in each ecosystem. Indeed, we cannot verify this assumption because we only placed glass slides in the grassland. Regardless of the dispersal treatment, however, ecosystem type was the main factor determining microbial community composition on the leaf litter, confirming previous results from this field site (38). Given that the grassland and CSS experience similar climate conditions, we attribute these ecosystem effects to differences in the leaf litter chemistry of the plant communities. The effect of ecosystem indicates that, in addition to dispersal, habitat filtering (selection) on the leaf litter communities or their dispersal sources is an important driver of microbial community succession.

Within ecosystems, dispersal impacted some aspects of bacterial and fungal community succession in a similar manner, including α-diversity and grassland β-diversity trends (Q2). This result countered our hypothesis that differences in traits, such as size, morphology, and dispersal modes of bacteria and fungi, would influence their dispersal patterns and therefore the effects of their dispersal on microbial succession (54 – 56). The similar way in which dispersal impacted both communities was also surprising, given that we assayed the communities using different marker genes. These findings suggest that other factors are more important for post-fire succession of both bacteria and fungi. For instance, charred leaf litter was used in this experiment, which contains highly aromatic structures that resist decomposition (57, 58), potentially constraining the effect of dispersal on both bacterial and fungal communities. Further, bacterial and fungal diversity showed similar patterns on the glass slides, matching seasonal shifts in abiotic conditions (Fig. 3B and C). Thus, abiotic properties seem to influence the effects of dispersal more than specific trait differences between bacteria and fungi. Although we do not expect mycorrhizal fungi to make up a signification portion of the leaf litter community, we note that primer bias may influence our characterization of the fungal communities in the open and closed bags as the internal transcribed spacer (ITS)2 primer does not detect all arbuscular mycorrhizal fungi (59, 60). Further, we cannot exclude the possibility that undetected differences in moisture or differences in the composition of small grazers, microfauna, and plant roots may also contribute to the successional differences seen between the communities exposed to dispersal and those that were not.

Taken together, our results demonstrate how dispersal explicitly contributes to bacterial and fungal succession following a wildfire. Previous work in this system shows that relatively minor shifts in microbial taxonomic composition can affect leaf litter decomposition rates (61) so the role of dispersal in post-fire succession could have consequential impacts on ecosystem processes such as carbon cycling. With other growing evidence that microbial communities are dispersal limited, future studies might aim to directly measure the functional consequences of dispersal. Further exploring whether more active management of key dispersal sources may expedite community recovery of soil microbial communities should also be considered.

## MATERIALS AND METHODS

### Field site and Silverado Fire

This experiment was conducted adjacent to the Loma Ridge Global Change Experiment in a California grassland and neighboring CSS located in northern Irvine, CA, USA (33°44′ N, 117°42′ W, 365-m elevation). Plant community composition varies between the grassland and CSS at Loma Ridge (38). The grassland is dominated by non-native annual grasses (*Bromus diandrus* and *Avena fatua*) and the native forb *Deinandra fasciculata*, while native drought-deciduous shrubs (*Artemisia californica* and *Salvia mellifera*) dominate the neighboring CSS (62, 63). Leaf litter chemistry also varies between the grassland and CSS. In particular, the shrub litter has higher lignin and lower cellulose content than that in the grassland and is more resistant to microbial decomposition (51, 64). In both ecosystems, leaf litter bacterial communities are dominated by the phyla *Proteobacteria* and *Actinobacteria*, and fungal communities are dominated by *Ascomycota* and *Basidiomycota* (38, 39). Soils are fine-loamy, mixed, thermic *Typic Palexeralfs* sandy loams (California Soil Resource Lab, https://casoilresource.lawr.ucdavis.edu/gmap/). In the top 15 cm of soil, total organic carbon pools are similar between the grassland and CSS, while total nitrogen is higher in the CSS (65). The climate is Mediterranean (dry summers and wet winters) with a mean annual temperature of 17°C and a mean annual precipitation of 325 mm.

On 26 October 2020, the grassland and CSS were burned by the Silverado Fire (Fig. 1). The wildfire reduced vegetation cover in both ecosystems. Fire intensity was not quantitatively assessed due to the unplanned nature of the fire and safety concerns preventing access to the site immediately after the fire. In both ecosystems, the fire removed most of the surface litter layer; however, some partially burned leaf litter remained on the soil surface. Partially charred leaf litter was collected in the grassland and CSS as soon as we were permitted into the site on 18 November 2020, 23 days after the wildfire (Fig. 1B and E insets).

### Dispersal manipulations

To manipulate microbial dispersal, litterbags were constructed from either 0.22-µm nylon or 2-mm window screen. The 2-mm pores in the window screen mesh allow bacterial and fungal cells to disperse in and out of the bags (“open” litterbags). Conversely, 0.22-µm pores in the nylon restrict immigration of bacteria and fungi (closed litterbags). Autoclaved litterbags (10 cm × 10 cm) were filled with 3 g of charred leaf litter (wet weight) collected from either the grassland or CSS (open litterbags: *n* = 35 per ecosystem, closed litterbags: *n* = 35 per ecosystem). Filled litterbags were stored at 4°C for up to 6 days.

To characterize the abundance and composition of the dispersal assemblage, 50 dispersal bags (5 cm × 7.5 cm) were filled with a single glass microscope slide (open: *n* = 35, closed: *n* = 15). Walters et al. (37) showed the closed bag treatment successfully prevents the glass slides from capturing dispersing microbes. Thus, we reduced the number of closed glass slides that we deployed into the field to minimize resource consumption and preparation time. Glass microscope slides (2.5 cm × 7.5 cm) were cleaned with diH_2_O, sterilized with 70% ethanol, dried, sealed into dispersal bags, and autoclaved.

On 25 November 2020, 30 days after the wildfire, the 70 grass and 70 CSS litterbags were deployed onto the soil surface of their respective ecosystems in 14 experimental blocks (1 m × 1 m, seven blocks per ecosystem). At the time of deployment, dispersal bags were placed directly onto exposed bulk soil, which had a thin, but heterogeneous, char layer still present (Fig. S1). Previous work at this site revealed that dispersal from vegetation contributes to the assembly of undisturbed leaf litter microbial communities (37). Due to the opportunistic nature of this experiment and limited resources, we kept the number of samples manageable and chose to only deploy the 50 glass slide dispersal bags into the grassland (Fig. 1F).

### Dispersal bag collection

At five timepoints, we collected 7 litterbags per dispersal treatment (2 ecosystems × 2 dispersal treatments × 7 replicates = 28 litterbags/timepoint), 7 open glass slides, and 3 closed glass slides (10 glass slide bags/timepoint). Dispersal bags were collected approximately 3 months (*T*1: 13 January 2021), 4 months (*T*2: 16 February 2021), 7 months (*T*3: 26 May 2021), 11 months (*T*4: 21 September 2021), and 15 months (*T*5: 11 January 2022) after the wildfire. We anticipated that dispersal would have a greater influence over community assembly immediately following the wildfire disturbance. We, therefore, concentrated collection timepoints toward the beginning of the experiment.

On the day of collection, open leaf litter and glass slide bags were placed in sterile plastic bags in the field before being transported back to the lab. Leaf litter samples were immediately ground with a coffee grinder and homogenized. A 0.1-g aliquot of ground leaf litter was placed into a 50-mL conical tube with 5 mL 1% phosphate-buffered glutaraldehyde (Pi-buffered GTA) and stored in the dark at 4°C for up to 2 days in preparation for bacterial abundance analysis. At each timepoint, moisture content was measured on a 1-g subsample of ground, homogenized leaf litter. Overall, leaf litter moisture content was not significantly different between the open and closed litter bags (*t*-test: *P* = 0.60). All remaining ground, homogenized leaf litter was stored at −70°C until DNA extraction.

Glass slides were transferred on collected day from dispersal bags into sterilized Whirl-Pak bags (Nasco, WI, USA) containing 2 mL 0.9% sterile saline. Notably, glass slides had a visible layer of dust and/or bulk soil on the surface at the time of collection and were often in direct contact with vegetation or leaf litter at later timepoints as the plant community recovered post-fire. The Whirl-Paks were agitated by hand for 30 s to dislodge microbial cells from the glass slide surface into the saline solution. A 600-µL aliquot of this cell solution was stored at −70°C until DNA extraction. Ten percent Pi-buffered GTA (156 µL) was added to the remaining cell solution (final concentration of 1% Pi-buffered GTA) and fixed samples were stored in the dark at 4°C for up to 12 h for bacterial abundance analysis.

### Dispersal source sampling

Surrounding air, bulk soil, and vegetation were previously identified as potential sources of dispersal into the surface leaf litter layer at this field site (37). At each sampling timepoint, we collected air (*n* = 2), surface soil (*n* = 1), and environmental (not litterbag) leaf litter samples (*n* = 1) from each ecosystem. To collect air samples, we directed airflow from a portable fan (O2Cool FD10101) at two sterile agar plates for 30 min. Air samples were collected 3 feet off the soil surface and within 10 feet of the experimental blocks in both ecosystems. On the day of collection, a sterile razor blade was used to scrape off the top 1 mm of agar. Environmental leaf litter and bulk soil samples were collected randomly from the seven experimental blocks and pooled into one composite sample for each timepoint. Soil samples were collected by scraping a sterile garden trowel across the soil surface to collect the top 1 cm of bulk soil. On the day of collection, soil samples were sieved (2 mm), and environmental leaf litter samples were ground and homogenized. Agar, soil, and environmental litter samples were stored at −70°C until DNA extraction.

### Bacterial abundance using flow cytometry

Bacterial abundance from grass litter, CSS litter, and glass slide samples was measured using flow cytometry (66). For grass litter samples, 550 µL 0.1 M tetrasodium pyrophosphate was added to the fixed sample and gently sonicated for 30 min in the dark at 4°C. The samples were then vacuum filtered through a 2.7-µm filter to remove larger non-bacterial cells and debris. GTA-fixed glass slide samples were also filtered through a 2.7-µm filter. As for all steps of microbial characterization, the ease in which cells dislodge from the glass slides may vary between taxa, potentially biasing downstream analyses.

Due to increased background noise created by debris particles, an optimized method to quantify bacterial abundance from soil and shrub leaf litter was used to prepare CSS litter samples for flow cytometry (66). To extract bacterial cells from the CSS litter, a detergent solution consisting of 1.2 mL 250 mM tetrasodium pyrophosphate (TSP) and 31 µL Tween 80 was added to the fixed samples followed by 30 min of gentle sonication in the dark at 4°C. One milliliter aliquots of the liquid slurry were then layered on top of 0.5 mL Nycodenz (80% [wt/vol] prepared in 50 mM sterile TSP buffer). Samples were then centrifuged for 30 min at 14,000 × *g*. The upper and middle cell-containing phases were collected and transferred to 1 mL 50 mM TSP followed by 25 min of centrifuging at 17,000 × *g*. The cell pellet was then resuspended in 800 µL 50 mM TSP.

All samples were processed through the flow cytometer on the day of filtration or isolation. To measure bacterial abundance on a NovoCyte flow cytometer (ACEA Biosciences, San Diego, CA, USA), 3 µL of 200× SYBR green (Invitrogen Life Science Technologies, S756, Grand Island, NY, USA) was added to the 600-µL final sample and incubated in the dark at room temperature for 15 min. Samples were run for 30 s at 40 µL/min. Flow cytometer gating parameters used to count cells were previously optimized (66). Cell abundance was calculated as the number of stained counts minus stained counts from control samples per gram dry litter or per square centimeter glass slide for leaf litter and glass slide samples, respectively.

Glass slide samples closed to dispersal had few cells (4,353 cells/cm^2^ on average) compared to the open glass slides (202,677 cells/cm^2^ on average), demonstrating that the closed bags effectively reduced dispersal. Given that these samples had such low abundance, DNA was not extracted nor sequenced from the closed glass slide bags, and we only report the results from the slides exposed to dispersal.

### DNA extraction and sequencing

Genomic DNA was extracted from 0.05 g ground litter, 0.1 g sifted soil, 250 µL unfiltered glass slide solution, and 0.05 to 0.1 g agar using ZymoBIOMICS 96 DNA Kits following the manufacturer’s protocol, except the maximum centrifuge force was 2,808 × *g*, instead of 3500 × *g*. For all leaf litter and soil samples, bead-beating was conducted for 5 min at 6.5 m/s in a FastPrep 24 (MP Biomedicals, Irvine CA, USA). Bead-beating was reduced to 3 min at 6.5 m/s for glass slide and air samples to avoid shearing DNA in these low-biomass samples. To minimize batch differences, all samples were randomized prior to DNA extraction.

To characterize bacterial community composition, we amplified the V4–V5 region of the 16S rRNA gene using the 515F (GTGYCAGCMGCCGCGGTAA) and 926R (CCGTCAATTCCTT TRAGTTT) primers (67, 68). For 16S PCRs, 1 µL genomic DNA was combined with 10.5 µL PCR grade water, 12.5 µL AccustartII PCR tough mix (Quanta BioSciences Inc, Beverly, MA, USA), 0.5 µL of the 10 µM barcoded forward primer, and 0.5 µL of the 10 µM reverse primer. For glass slide and air samples, 5 µL genomic DNA was added with only 6.5 µL PCR grade water. An initial denaturation step was performed at 94°C for 3 min, followed by 30 cycles of denaturing at 94°C for 45 s, annealing at 55°C for 30 s, and extension at 72°C for 60 s, with a final extension at 72°C for 10 min.

To characterize fungal community composition, we amplified the ITS2 region using ITS9F (GAACGCAGCRAAIIGYGA) and ITS4R (TCCTCCGCTTATTGATATGC) primers (69). For ITS PCR reactions, 1 µL genomic DNA was combined with 10 µL PCR grade water, 12.5 µL AccustartII PCR tough mix (Quanta BioSciences Inc), 0.75 µL of the 10 µM barcoded forward primer, and 0.75 µL of the 10 µM reverse primer. For glass slide and air samples, 5 µL genomic DNA was added with only 6 µL PCR grade water. An initial denaturation step was performed at 94°C for 5 min, followed by 35 cycles of denaturing at 95°C for 45 s, annealing at 50°C for 60 s, and extension at 72°C for 90 s, with a final extension at 72°C for 10 min.

Sequencing libraries were created by pooling PCR products based on band brightness in gel pictures (high [1 µL], medium [2 µL], and low [3 µL], very low [5 µL], and no band [8 µL]). Originally, the 16S and ITS amplicons from the experimental litter, environmental litter, and environmental soil samples were pooled together in one library, and amplicons from the experimental glass slide and environmental air samples were pooled together in a second library. Both libraries were cleaned using Sera-Mag SpeedBeads (70). The amplicon libraries were sequenced separately in two paired-end Illumina MiSeq (2 × 300 bp) runs by the UC Irvine Genomics High Throughput Sequencing Facility (Irvine, CA, USA). Due to poor sequencing quality, the 16S amplicons from all samples were repooled, cleaned, and sequenced in a separate run. Low sequencing reads were obtained again for CSS leaf litter samples from the first and second collection dates (January and February 2021). Thus, DNA from 16 of these samples with poor sequencing results and 7 samples that sequenced well in previous runs were reextracted, reamplified, and resequenced in a third sequencing run.

### Amplicon sequencing processing

Forward reads from the three Illumina amplicon libraries were demultiplexed separately using QIIME2, version 2021.2 (71). Reverse reads were discarded from all runs due to low sequencing quality. Forward reads were trimmed to 237 bases, and DADA2 was used to define operational taxonomic units (OTUs) defined at 100% identity (sequence variants) for all three libraries (72). Trimmed and denoised sequences from all independent MiSeq runs were then merged to create a single OTU table. Taxonomic identity was assigned using the q2-feature-classifer plugin and classify-sklearn in QIIME2 (73) to generate a Naïve Bayes classifier trained on reference sequences from the SILVA 138 SSU Ref NR99 database (74) filtered at 99% identity trimmed to 237 bp for bacteria and untrimmed UNITE database version 8.3 for fungi (75). Sequences assigned to chloroplast, mitochondria, Archaea, or unidentified at the phylum level were removed prior to downstream analysis.

To compare our post-fire leaf litter communities with pre-fire samples, we reprocessed 16S and ITS amplicon sequences obtained from a previous leaf litter survey conducted from August 2016 to March 2018 at this field site (38). Forward reads from the pre-fire library were trimmed to 237 bases, denoised, and merged with the post-fire sequences to create a separate OTU table. Taxonomic identity was then assigned using the same SILVA and UNITE classifiers as previously mentioned.

### Statistical analysis

To account for differences in sequencing depth among samples, we rarefied OTU tables produced in QIIME2 to 1,300 sequences or 1,328 sequences for the bacterial and fungal communities, respectively, with 300 resamplings using the EcolUtilis package in R version 4.0.3 (76, 77). Community composition was compared between samples using Bray-Curtis dissimilarity matrices generated from square root transformed rarefied OTU tables. To assess how the composition of dispersing microbes changed across time and test the effects of dispersal on leaf litter microbial community composition following the wildfire, permutational multivariate analysis of variance (PERMANOVA) and post hoc tests were performed using PERMANOVA+ on PRIMER version 6 (40, 78). Block was included as a random effect factor for all PERMANOVA models. All PERMANOVA analyses were run as type III partial sum of squares for 999 permutations. Variance explained by each experimental variable was calculated by dividing the estimated components of variance of statistically significant terms by the sum of all significant terms and the residuals. The proportion of the glass slide communities attributed to different dispersal sources (air, environmental leaf litter, and bulk soil) was estimated using SourceTracker (version 1.0.1) in R with default parameters, except alpha1 and alpha2 were tuned to 0.001 and 0.1, respectively, for the bacterial community analysis using cross-validation and 0.001 for both parameters for the fungal community analysis (79).

Given that microbial dispersal can influence β-diversity and PERMANOVA is sensitive to differences in dispersion, we ran pairwise comparisons of group mean dispersions between the dispersal treatments using PERMDISP on PERMANOVA+. To quantify the variation in community composition within open and closed bags from both ecosystems, we assessed the distance of each sample to the group centroid using the “distance among centroids” function in PERMANOVA+ (40). Non-metric multidimensional scaling ordination plots were generated from the Bray-Curtis dissimilarity matrices to visualize the effect of dispersal on microbial composition and β-diversity (dispersion). Finally, a SIMPER analysis was performed in PRIMER version 6 (78) to distinguish which genera contributed most to the compositional differences between leaf litter and glass slide communities as well as the burned and unburned samples.

To test for differences in univariate metrics (α-diversity and bacterial abundance) between dispersal treatments and across time, mixed model analysis of variance (ANOVA) was performed using the “lmer” function from the lme4 package in R (80). Experimental block was included as a repeated-measure, random effect. The repeated measures mixed model ANOVAs took the general form of (univariate metric) ~ (bag_type) × (timepoint) + (1|block) for the leaf litter samples and (univariate) ~ (timepoint) + (1|block) for the glass slide samples. These model designs account for non-independence within blocks and repeated measures across time. Significant pairwise comparisons were determined using post hoc Tukey’s honestly significant difference test. The Shannon diversity index and observed OTU richness were highly correlated for both fungal and bacterial communities from the glass slide and leaf litter samples (Spearman’s correlation: *P* < 0.001 in all cases). Therefore, we chose to only report results for Shannon diversity.

## Data Availability

The raw amplicon reads are available through the NCBI Sequence Read Archive under BioProject accession number PRJNA973138.

## SUPPLEMENTAL MATERIAL

Supplemental material msystems.00579-23-s0001.docx Supplemental figures and tables.

ASM does not own the copyrights to Supplemental Material that may be linked to, or accessed through, an article. The authors have granted ASM a non-exclusive, world-wide license to publish the Supplemental Material files. Please contact the corresponding author directly for reuse.
