# Supplementary material for: Testing the contribution of dispersal to microbial succession following a wildfire
Source: mSystems. 2023 Sep 25;8(5):e00579-23. doi: 10.1128/msystems.00579-23 (PMC10654055; doi:10.1128/msystems.00579-23)
Supplement: Supplemental material — Supplemental figures and tables. [file msystems.00579-23-s0001.docx]

**Supplementary Figures**

**
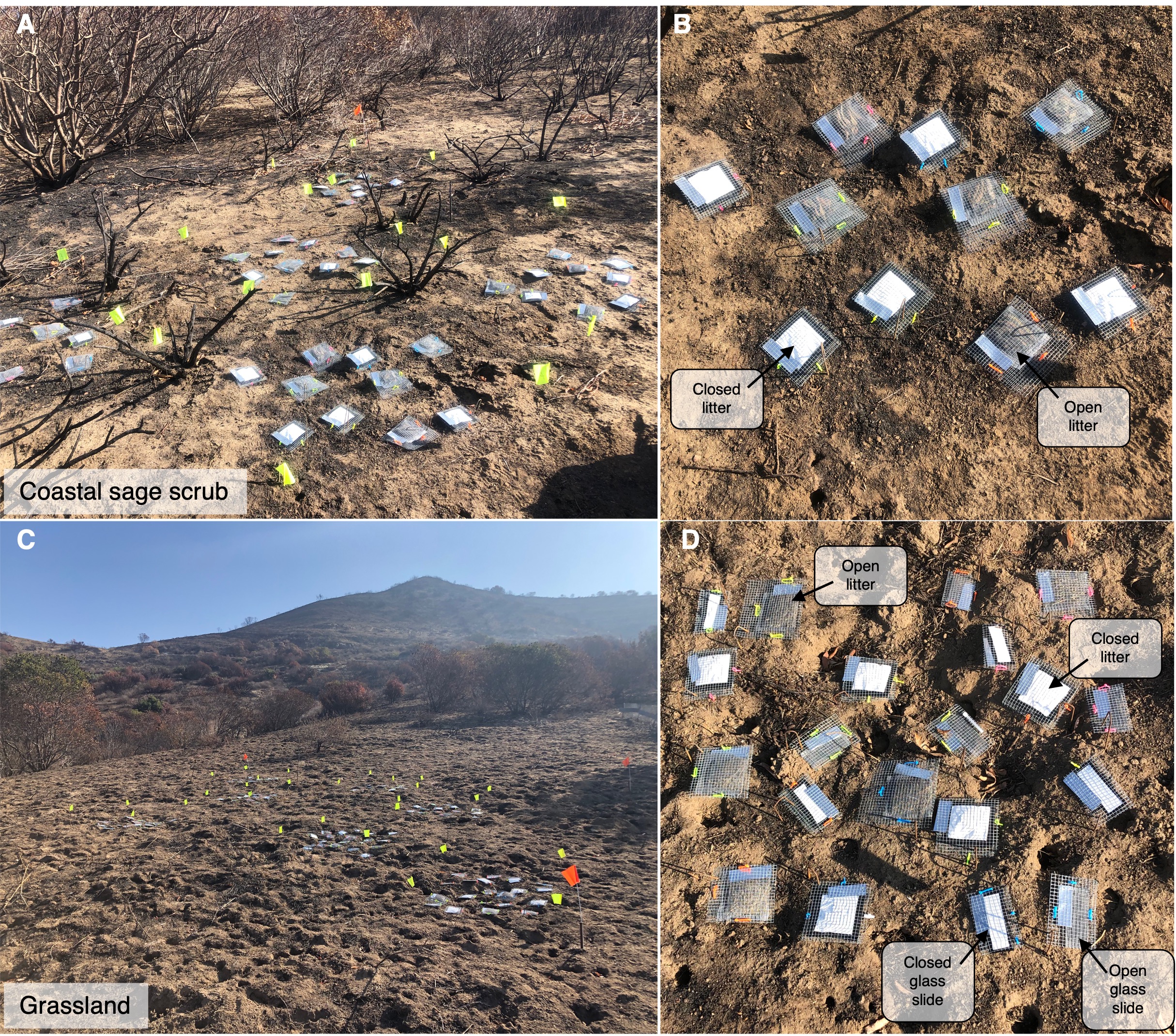
**

Fig. S1. (A) Distribution of dispersal bags in the CSS at Loma Ridge at the time of deployment into the field. Yellow flags mark the four corners of each experimental block (n = 7). (B) One experimental block in the CSS illustrating the randomized placement of dispersal bags on the soil surface. Labels indicate the open and closed leaf litter treatments. (C) Distribution of dispersal bags in the grassland at Loma Ridge at the time of deployment into the field. Yellow flags mark the four corners of each experimental block (n = 7). (B) One experimental block in the grassland illustrating the randomized placement of leaf litter and glass slide dispersal bags on the soil surface. Labels indicate the open and closed leaf litter and glass slide treatments. (A-D) At time of deployment, all bags were placed onto the exposed bulk soil on the soil surface. Char was still visible on the soil surface but heterogenous across the landscape as depicted.

Fig. S2. Relative abundance of (A) bacterial and (B) fungal genera dispersing onto the glass slides. “Other” genera represent all classified genera below 3% relative abundance.

Fig S3. Composition of (A) bacterial and (B) fungal genera from different dispersal sources (air, environmental litter, and surface soil) within the grassland and CSS. “Other” genera represent all classified genera below 3% relative abundance.

Fig. S4. Estimated percent variation explained for significant factors from mixed-effects PERMANOVAs for bacterial and fungal communities within all leaf litter, CSS leaf litter, grassland leaf litter, and glass slide samples.


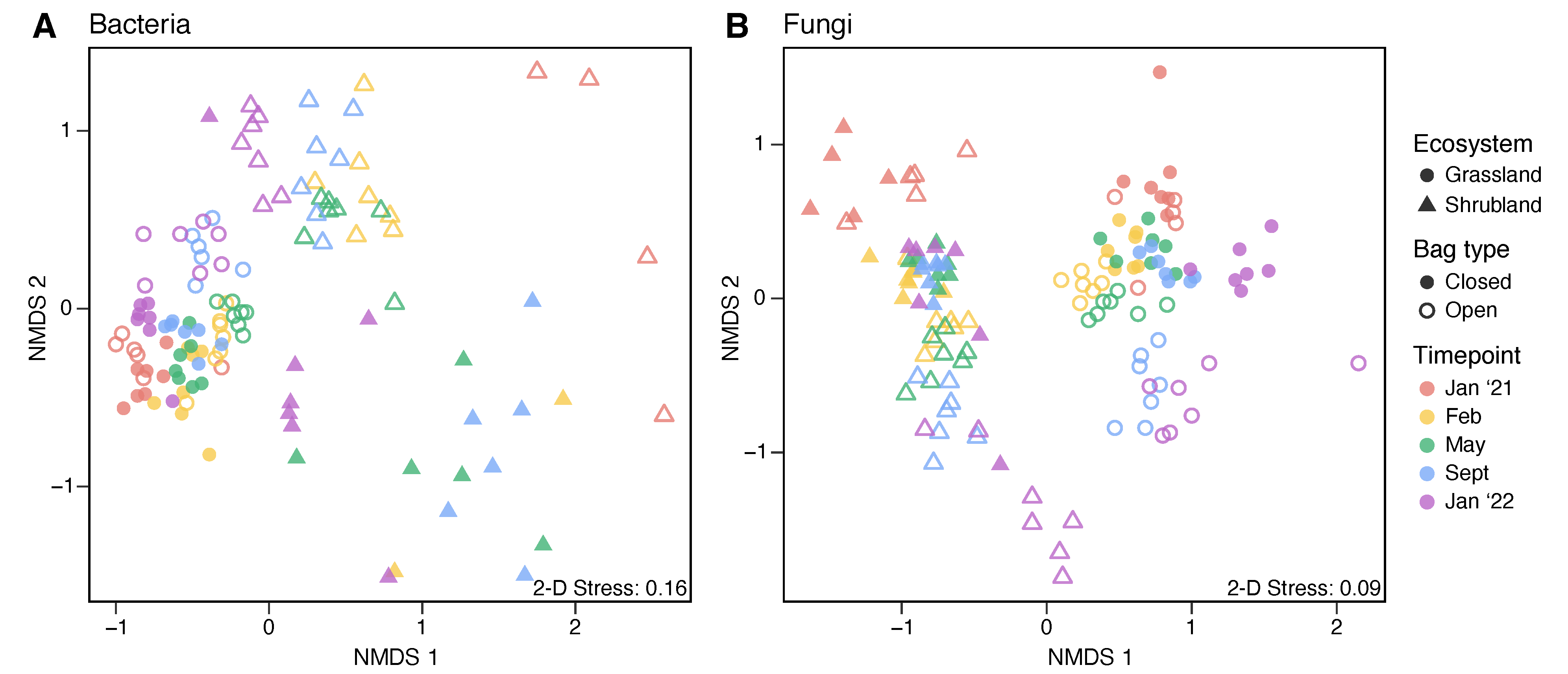


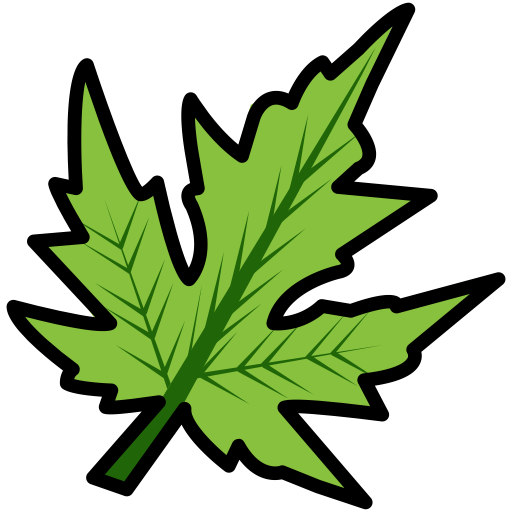

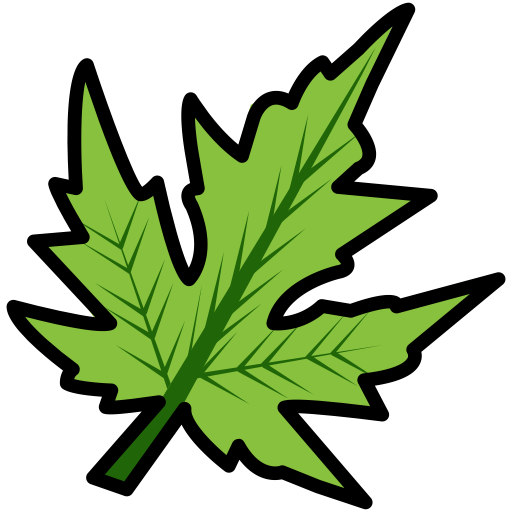

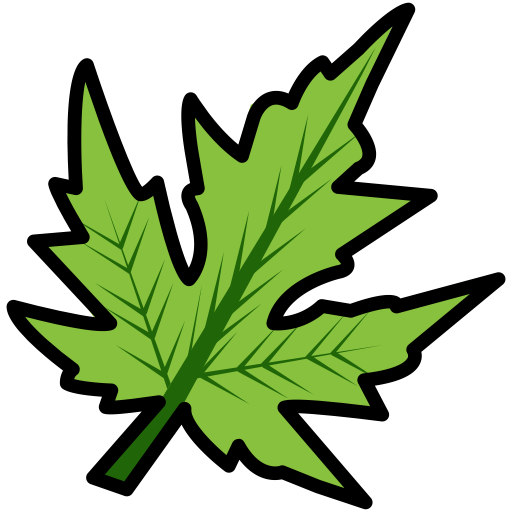

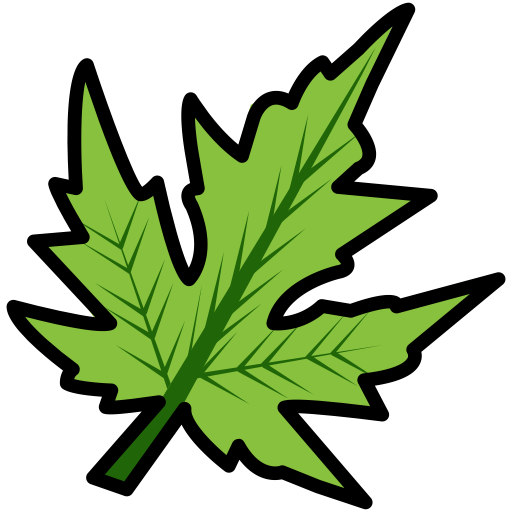

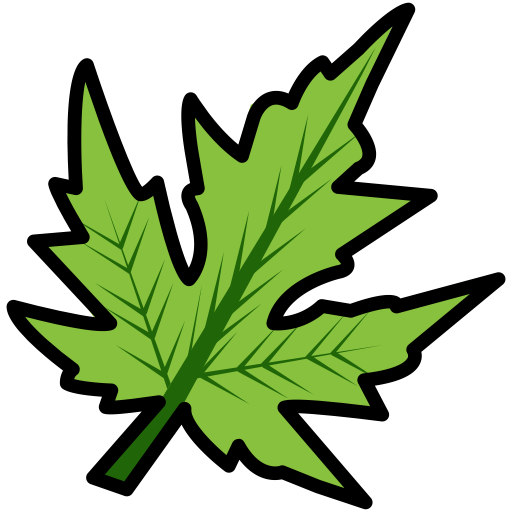

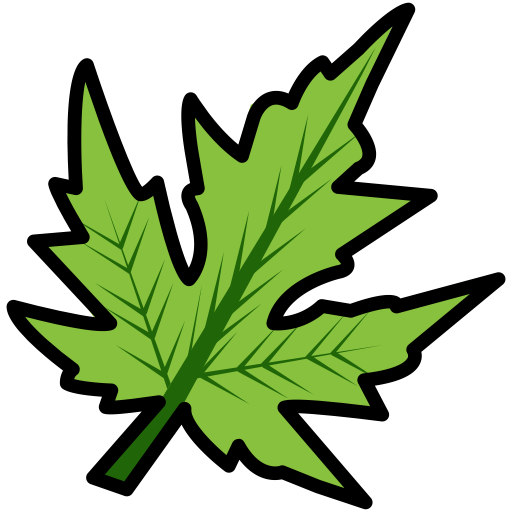


Fig. S5. Non-metric multidimensional scaling (NMDS) ordination of leaf litter (A) bacterial and (B) fungal community composition. Symbol shape represents ecosystem type (circle = grassland, triangle = CSS) and dispersal bag type (filled = closed, outline = open). Symbol color represents collection timepoint.

**Supplementary tables**

Table S1. SIMPER analysis showing the top 3 classified bacterial and fungal genera contributing to differences between the glass slide (dispersal community) and burned leaf litter from the grassland only at Loma Ridge.

| SIMPER Analysis | | |
| --- | --- | --- |
| Genus | Greater mean abundance | % Contribution |
| **Bacteria** |  |  |
| *Hymenobacter* | Glass slides | 4.31 |
| *Massilia* | Burned litter | 3.06 |
| *Sphingomonas* | Burned litter | 2.92 |
|  |  |  |
| **Fungi** |  |  |
| *Filobasidium* | Glass slides | 4.43 |
| *Coniochaeta* | Burned litter | 3.39 |
| *Alternaria* | Glass slides | 3.27 |

Table S2. ﻿ANOVA analysis of bacterial abundance from all leaf litter, grassland leaf litter, CSS leaf litter, and glass slide samples separately. Significant factors and p-values are bolded.

| ANOVA  **Bacterial abundance** | | | | | |
| --- | --- | --- | --- | --- | --- |
| All leaf litter samples |  |  |  |  |  |
| Factor(s) | df | SS | MS | Pseudo-F | P |
| **Ecosystem** | **1** | **171.10** | **171.10** | **1373.24** | **< 2.20E-16** |
| **Ecosystem x Bag type** | 1 | 4.09 | 4.09 | 32.83 | **7.62E-08** |
| **Ecosystem x Timepoint** | 4 | 6.93 | 1.73 | 13.90 | **2.39E-09** |
| **Ecosystem x Bag type x Timepoint** | 4 | 1.28 | 0.32 | 2.56 | **0.04** |
| Bag type | 1 | 0.36 | 0.36 | 2.89 | 0.09 |
| **Bag type x Timepoint** | 4 | 1.47 | 0.37 | 2.94 | **0.02** |
| **Timepoint** | 4 | 5.91 | 1.48 | 11.86 | **3.75E-08** |
|  |  |  |  |  |  |
| Grassland leaf litter samples |  |  |  |  |  |
| Factor(s) | df | SS | MS | Pseudo-F | P |
| **Bag type** | 1 | 1.01 | 1.01 | 29.16 | **1.20E-06** |
| Bag type x Timepoint | 4 | 0.18 | 0.05 | 1.31 | 0.28 |
| **Timepoint** | 4 | 0.54 | 0.13 | 3.89 | **0.01** |
|  |  |  |  |  |  |
| CSS leaf litter samples |  |  |  |  |  |
| Factor(s) | df | SS | MS | Pseudo-F | P |
| **Bag type** | 1 | 3.44 | 3.44 | 16.03 | **1.74E-04** |
| **Bag type x Timepoint** | 4 | 2.56 | 0.64 | 2.99 | **0.03** |
| **Timepoint** | 4 | 12.30 | 3.08 | 14.34 | **2.85E-08** |
|  |  |  |  |  |  |
| Glass slide samples |  |  |  |  |  |
| Factor(s) | df | SS | MS | Pseudo-F | P |
| **Timepoint** | 4 | 11.99 | 3.00 | 16.63 | **1.18E-06** |

Table S3. ﻿ANOVA analysis of bacterial Shannon diversity index from all leaf litter, grassland leaf litter, CSS leaf litter, and glass slide samples separately. Significant factors and p-values are bolded.

| ANOVA  **Bacterial Shannon diversity index** | | | | | |
| --- | --- | --- | --- | --- | --- |
| All litter samples |  |  |  |  |  |
| Factor(s) | df | SS | MS | F-value | P |
| **Ecosystem** | 1 | 36.38 | 36.38 | 250.55 | **< 2.20E-16** |
| **Ecosystem x Bag type** | 1 | 25.52 | 25.52 | 175.72 | **< 2.20E-16** |
| **Ecosystem x Timepoint** | 4 | 10.36 | 2.59 | 17.84 | **4.15E-11** |
| Ecosystem x Bag type x Timepoint | 3 | 0.95 | 0.32 | 2.19 | 0.09 |
| **Bag type** | 1 | 17.29 | 17.29 | 119.10 | **< 2.20E-16** |
| **Bag type x Timepoint** | 4 | 1.59 | 0.40 | 2.74 | **0.03** |
| **Timepoint** | 4 | 10.83 | 2.71 | 18.64 | **1.68E-11** |
|  |  |  |  |  |  |
| Grassland litter samples |  |  |  |  |  |
| Factor(s) | df | SS | MS | F-value | P |
| **Bag type** | 1 | 0.47 | 0.47 | 6.03 | **0.02** |
| **Bag type x Timepoint** | 4 | 2.13 | 0.53 | 6.89 | **1.31E-04** |
| **Timepoint** | 4 | 3.51 | 0.88 | 11.36 | **7.08E-07** |
|  |  |  |  |  |  |
| CSS litter samples |  |  |  |  |  |
| Factor(s) | df | SS | MS | F-value | P |
| **Bag type** | 1 | 34.83 | 34.83 | 149.90 | **4.27E-15** |
| Bag type x Timepoint | 3 | 0.87 | 0.29 | 1.25 | 0.30 |
| **Timepoint** | 4 | 17.50 | 4.37 | 18.83 | **1.10E-08** |
|  |  |  |  |  |  |
| Glass slide samples |  |  |  |  |  |
| Factor | df | SS | MS | F-value | P |
| **Timepoint** | 4 | 22.43 | 5.61 | 19.87 | **4.39E-08** |

Table S4. ﻿PERMANOVA analysis of bacterial communities from all leaf litter, grassland leaf litter, CSS leaf litter, and glass slide samples separately. Significant factors and p-values are bolded.

| PERMANOVA (Bacteria**)** | | | | | | |
| --- | --- | --- | --- | --- | --- | --- |
| All leaf litter samples |  |  |  |  |  |  |
| Factor(s) | df | SS | MS | Pseudo-F | P | % Variance Explained |
| **Ecosystem** | 1 | 43434 | 43434 | 42.31 | **0.001** | 26.43 |
| **Ecosystem x Bag type** | 1 | 14074 | 14074 | 15.40 | **0.001** | 14.33 |
| **Ecosystem x Timepoint** | 4 | 19950 | 4987.5 | 5.46 | **0.001** | 9.98 |
| **Ecosystem x Bag type x Timepoint** | 3 | 5619 | 1873.1 | 2.05 | **0.001** | 4.02 |
| **Bag type** | 1 | 19808 | 19808 | 21.68 | **0.001** | 10.47 |
| **Bag type x Timepoint** | 4 | 9262 | 2315.4 | 2.53 | **0.001** | 3.31 |
| **Timepoint** | 4 | 31549 | 7887.2 | 8.63 | **0.001** | 8.48 |
| **Block** | 12 | 12983 | 1081.9 | 1.18 | **0.004** | 0.49 |
| Residuals | 89 | 81325 | 913.76 |  |  | 22.50 |
| Total | 119 | 258680 |  |  |  |  |
|  |  |  |  |  |  |  |
| Grassland leaf litter samples |  |  |  |  |  |  |
| Factor(s) | df | SS | MS | Pseudo-F | P | % Variance Explained |
| **Bag type** | 1 | 9272 | 9271.9 | 14.88 | **0.001** | 17.69 |
| **Bag type x Timepoint** | 4 | 24242 | 6060.6 | 9.73 | **0.001** | 11.48 |
| **Timepoint** | 4 | 6984 | 1746 | 2.80 | **0.001** | 27.79 |
| Block | 6 | 4267 | 711.11 | 1.14 | 0.081 | - |
| Residuals | 52 | 32404 | 623.16 |  |  | 43.05 |
| Total | 67 | 77351 |  |  |  |  |
|  |  |  |  |  |  |  |
| CSS leaf litter samples |  |  |  |  |  |  |
| Factor(s) | df | SS | MS | Pseudo-F | P | % Variance Explained |
| **Bag type** | 1 | 24292 | 24292 | 18.37 | **0.001** | 34.29 |
| **Bag type x Timepoint** | 4 | 30858 | 7714.6 | 5.83 | **0.001** | 7.37 |
| **Timepoint** | 3 | 8075 | 2691.7 | 2.04 | **0.001** | 20.31 |
| Block | 6 | 8717 | 1452.8 | 1.10 | 0.141 | - |
| Residuals | 37 | 48920 | 1322.2 |  |  | 38.04 |
| Total | 51 | 124170 |  |  |  |  |
|  |  |  |  |  |  |  |
| Glass slide samples |  |  |  |  |  |  |
| Factor(s) | df | SS | MS | Pseudo-F | P | % Variance Explained |
| **Timepoint** | 4 | 30509 | 7627.2 | 4.45 | **0.001** | 32.41 |
| **Block** | 6 | 11683 | 1947.2 | 1.14 | **0.020** | 1.78 |
| Residuals | 24 | 41164 | 1715.2 |  |  | 65.81 |
| Total | 34 | 83356 |  |  |  |  |

Table S5. ﻿ANOVA analysis of fungal Shannon diversity index from all leaf litter, grassland leaf litter, CSS leaf litter, and glass slide samples separately. Significant factors and p-values are bolded.

| ANOVA  **Fungal Shannon diversity index** | | | | | |
| --- | --- | --- | --- | --- | --- |
| All litter samples |  |  |  |  |  |
| Factor(s) | df | SS | MS | F-value | P |
| **Ecosystem** | 1 | 39.32 | 39.32 | 488.94 | **<2.20E-16** |
| **Ecosystem x Bag type** | 1 | 1.70 | 1.70 | 21.12 | **1.12E-05** |
| **Ecosystem x Timepoint** | 4 | 5.09 | 1.27 | 15.82 | **2.51E-10** |
| **Ecosystem x Bag type x Timepoint** | 4 | 2.29 | 0.57 | 7.10 | **3.83E-05** |
| **Bag type** | 1 | 0.91 | 0.91 | 11.31 | **0.001** |
| Bag type x Timepoint | 4 | 0.31 | 0.08 | 0.96 | 0.43 |
| **Timepoint** | 4 | 2.77 | 0.69 | 8.61 | **4.12E-06** |
|  |  |  |  |  |  |
| Grassland litter samples |  |  |  |  |  |
| Factor(s) | df | SS | MS | F-value | P |
| Bag type | 1 | 0.06 | 0.06 | 0.68 | 0.41 |
| **Bag type x Timepoint** | 4 | 1.71 | 0.43 | 4.70 | **0.002** |
| Timepoint | 4 | 0.87 | 0.22 | 2.38 | 0.06 |
|  |  |  |  |  |  |
| CSS litter samples |  |  |  |  |  |
| Factor(s) | df | SS | MS | F-value | P |
| **Bag type** | 1 | 2.50 | 2.50 | 36.07 | **1.49E-07** |
| **Bag type x Timepoint** | 4 | 0.90 | 0.22 | 3.23 | **0.02** |
| **Timepoint** | 4 | 6.94 | 1.73 | 25.03 | **6.44E-12** |
|  |  |  |  |  |  |
| Glass slide samples |  |  |  |  |  |
| Factor | df | SS | MS | F-value | P |
| **Timepoint** | 4 | 53564 | 13391 | 44.14 | **3.39E-10** |

Table S6. ﻿PERMANOVA analysis of fungal communities from all leaf litter, grassland leaf litter, CSS leaf litter, and glass slide samples separately. Significant factors and p-values are bolded.

| PERMANOVA (Fungi) | | | | | | |
| --- | --- | --- | --- | --- | --- | --- |
| All leaf litter samples |  |  |  |  |  |  |
| Factor(s) | df | SS | MS | Pseudo-F | P | % Variance Explained |
| **Ecosystem** | 1 | 86990 | 86990 | 80.10 | **0.001** | 34.11 |
| **Ecosystem x Bag type** | 1 | 8584 | 8583.5 | 7.94 | **0.001** | 5.98 |
| **Ecosystem x Timepoint** | 4 | 20048 | 5012.1 | 4.64 | **0.001** | 7.73 |
| **Ecosystem x Bag type x**  **Timepoint** | 4 | 8567 | 2141.6 | 1.98 | **0.001** | 4.18 |
| **Bag type** | 1 | 19127 | 19127 | 17.69 | **0.001** | 7.19 |
| **Bag type x Timepoint** | 4 | 14354 | 3588.6 | 3.32 | **0.001** | 4.94 |
| **Timepoint** | 4 | 35426 | 8856.4 | 8.19 | **0.001** | 7.64 |
| Block | 12 | 13033 | 1086.1 | 1.00 | 0.473 | - |
| Residuals | 102 | 110280 | 1081.2 |  |  | 28.22 |
| Total | 133 | 321080 |  |  |  |  |
|  |  |  |  |  |  |  |
| Grassland leaf litter samples |  |  |  |  |  |  |
| Factor(s) | df | SS | MS | Pseudo-F | P | % Variance Explained |
| **Bag type** | 1 | 11247 | 11247 | 9.54 | **0.001** | 14.78 |
| **Bag type x Timepoint** | 4 | 9676 | 2419 | 2.05 | **0.001** | 9.06 |
| **Timepoint** | 4 | 24675 | 6168.7 | 5.23 | **0.001** | 18.22 |
| Block | 6 | 7387 | 1231.1 | 1.04 | 0.254 | - |
| Residuals | 52 | 61281 | 1178.5 |  |  | 57.95 |
| Total | 67 | 115240 |  |  |  |  |
|  |  |  |  |  |  |  |
| CSS leaf litter samples |  |  |  |  |  |  |
| Factor(s) | df | SS | MS | Pseudo-F | P | % Variance Explained |
| **Bag type** | 1 | 16348 | 16348 | 16.68 | **0.001** | 20.72 |
| **Bag type x Timepoint** | 4 | 13106 | 3276.5 | 3.34 | **0.001** | 15.19 |
| **Timepoint** | 4 | 30378 | 7594.5 | 7.75 | **0.001** | 21.78 |
| Block | 6 | 5646 | 941.07 | 0.96 | 0.678 | - |
| Residuals | 50 | 49002 | 980.05 |  |  | 42.31 |
| Total | 65 | 116950 |  |  |  |  |
|  |  |  |  |  |  |  |
| Glass slide samples |  |  |  |  |  |  |
| Factor(s) | df | SS | MS | Pseudo-F | P | % Variance Explained |
| **Timepoint** | 4 | 19567 | 4891.8 | 3.97 | **0.001** | 30.20 |
| **Block** | 6 | 9306 | 1550.9 | 1.26 | **0.012** | 3.66 |
| Residuals | 22 | 27121 | 1232.8 |  |  | 66.14 |
| Total | 32 | 56088 |  |  |  |  |

Table S7. SIMPER analysis showing the top 3 classified bacterial and fungal genera contributing to differences between the burned and unburned leaf litter at Loma Ridge.

| SIMPER Analysis | | |
| --- | --- | --- |
| Genus | Greater mean abundance | % Contribution |
| **Bacteria** |  |  |
| *Pseudomonas* | Burned | 3.84 |
| *Curtobacterium* | Unburned | 2.89 |
| *Pedobacter* | Burned | 2.31 |
| *Massilia* | Burned | 2.31 |
|  |  |  |
| **Fungi** |  |  |
| *Aureobasidium* | Burned | 6.22 |
| *Alternaria* | Unburned | 4.3 |
| *Cladosporium* | Burned | 3.38 |

Table S8. SIMPER analysis showing the top 3 classified bacterial and fungal genera contributing to differences between the open and closed dispersal treatments for the grassland and CSS litter independently.

| SIMPER Analysis | | |
| --- | --- | --- |
| Genus | Greater mean abundance | % Contribution |
| **Grassland litter** |  |  |
| **Bacteria** |  |  |
| *Massilia* | Open | 3.00 |
| *Pseudomonas* | Closed | 2.89 |
| *Hymenobacter* | Open | 2.87 |
|  |  |  |
| **Fungi** |  |  |
| *Coniochaeta* | Open | 5.88 |
| *Cladosporium* | Closed | 3.71 |
| *Aureobasidium* | Open | 3.33 |
|  |  |  |
| **CSS litter** |  |  |
| **Bacteria** |  |  |
| *Curtobacterium* | Closed | 5.87 |
| *Pseudomonas* | Closed | 4.83 |
| *Massilia* | Open | 3.55 |
|  |  |  |
| **Fungi** |  |  |
| *Cladosporium* | Closed | 9.14 |
| *Curvibasidium* | Open | 6.69 |
| *Naganishia* | Open | 5.14 |
